# A cross-sectional study of asymptomatic *Plasmodium falciparum* infection burden and risk factors in general population children in 12 villages in northern Uganda

**DOI:** 10.1186/s12936-018-2379-1

**Published:** 2018-06-20

**Authors:** Marlena Maziarz, Hadijah Nabalende, Isaac Otim, Ismail D. Legason, Tobias Kinyera, Martin D. Ogwang, Ambrose O. Talisuna, Steven J. Reynolds, Patrick Kerchan, Kishor Bhatia, Robert J. Biggar, James J. Goedert, Ruth M. Pfeiffer, Sam M. Mbulaiteye

**Affiliations:** 1 Division of Cancer Epidemiology and Genetics, National Cancer Institute, National Institutes of Health, 9609 Medical Center Dr, Rm. 6E118 MSC 9706, Bethesda, MD 20892-9704 USA; 2 EMBLEM Study, African Field Epidemiology Network, Kampala & St. Mary’s Hospital, Lacor, Gulu, Uganda; 3 World Health Organization, Regional Office for Africa, Brazzaville, Congo; 4 Division of Intramural Research, National Institute of Allergy and Infectious Diseases, National Institutes of Health, Bethesda, MD USA

**Keywords:** Burkitt lymphoma, Africa, *Plasmodium falciparum*, Malaria, Epidemiology, Non-Hodgkin lymphoma, Uganda

## Abstract

**Background:**

*Plasmodium falciparum* malaria is an important cause of morbidity in northern Uganda. This study was undertaken to assess village-, household-, and individual-level risk factors of asymptomatic *falciparum* malaria in children in 12 villages in northern Uganda.

**Methods:**

Between 10/2011 and 02/2014, 1006 apparently healthy children under 16 years old were enrolled in 12 villages using a stratified, multi-stage, cluster survey design and assessed for *P. falciparum* malaria infection using the rapid diagnostic test (RDT) and thick film microscopy (TFM), and structured interviewer-administered questionnaires. Associations between weighted *P. falciparum* malaria prevalence (pfPR), based on RDT, and covariates were estimated as odds ratios and 95% confidence intervals (ORs, 95% CIs) using logistic models accounting for the survey design.

**Results:**

Among 942 (93.5%) children successfully tested, pfPR was 52.4% by RDT and 32.7% by TFM. Overall pfPR was lower in villages where indoor residual insecticide spray (IRS) was, *versus* not, implemented (18.4% versus 75.2%, P < 0.0001). However, pfPR was heterogeneous both within IRS (10.6–34.8%) and non-IRS villages (63.6–86.2%). Elevated pfPR was associated with having a sibling who was RDT positive (OR 5.39, 95% CI 2.94–9.90, P = 0.0006) and reporting a fever at enrollment (aOR 4.80, 95% CI 1.94–11.9, P = 0.0094). Decreased pfPR was associated with living in an IRS village (adjusted OR 0.06, 95% CI 0.04–0.07, P < 0.0001), in a household with one (aOR 0.48, 95% CI 0.30–0.76) or more than one child below 5 years (aOR 0.23, 95% CI 0.12–0.44, P_trend_ = 0.014), and reporting keeping a goat inside or near the house (aOR 0.42, 95% CI 0.29–0.62, P = 0.0021).

**Conclusions:**

The results show high but heterogeneous pfPR in villages in northern Uganda, confirm significantly decreased pfPR associated with IRS implementation, and suggest significant associations with some household characteristics. Further research is needed to elucidate the factors influencing malaria heterogeneity in villages in Uganda.

**Electronic supplementary material:**

The online version of this article (10.1186/s12936-018-2379-1) contains supplementary material, which is available to authorized users.

## Background

*Plasmodium falciparum* malaria remains an important cause of morbidity and mortality globally, particularly in children aged less than 5 years old in countries in sub-Saharan Africa [1, 2], including Uganda [3]. Scale-up of malaria control interventions since the mid-2000s has resulted in significant declines in malaria morbidity and mortality in many countries [4–6]. However, the changes in malaria morbidity and mortality are heterogenous in different countries, characterized by increases or slower declines in some countries [7], and heterogenous changes in the incidence patterns in different geographical areas of the same country [8]. However, malaria surveillance is mostly based on acute malaria mortality and morbidity at health facilities, which may not correlate perfectly with impact on asymptomatic malaria infections, which comprise ≥ 75% of all infections [9], and represent an important source of new infections in a population [7]. A better understanding of the epidemiology of asymptomatic malaria in high-burden countries may help to design interventions to reduce the local burden and clinical impact of malaria [10].

Continuing intense *P. falciparum* malaria transmission has been reported in northern Uganda [11], an area home to some of the world’s highest malaria transmission rates (annual entomological inoculation rates of 400–1500 infectious mosquito bites per person, per year [12]). Between 2008 and 2015, the Ugandan Government, with the support of international donor partners, implemented 4–6 monthly indoor residual spraying (IRS) cycles and distribution of long-lasting insecticide-impregnated bed nets (LLIN) to the general population in 10 districts in northern Uganda [13, 14], leading to strong suppression of malaria prevalence in the targeted districts [13, 14]. The dramatic declines in outpatient malaria cases in children aged below 5 years old led to optimism that prompted withdrawal of IRS in 2014. However, this decision was premature because it was followed by a rapid increase of malaria cases [15]. The rapid reversal of trends in malaria morbidity following withdrawal of IRS in northern Uganda highlights the likely role of undetected subclinical infections in contributing to new cases of malaria [7]. The patterns of asymptomatic falciparum infection in northern Uganda before or after IRS are not well known. Most malaria data are based on national surveys, which are not sufficiently granular to provide local insights [16], focus on participants in a restricted age range, usually children below 5 years old [14], or on clinical cases [17], which provide limited information about the asymptomatic cases that represent the main reservoir of infection that spawns new clinical cases. This study was undertaken to obtain village- household-, and individual-level information about risk factors of asymptomatic malaria infection in children in northern Uganda, which would complement data based on broad sampling at a national [14, 16] and regional levels [13]. However, the study had an additional motivation: *P. falciparum* malaria is thought to be important in endemic Burkitt’s lymphoma (eBL) [18]. Thus, the study also aimed to generate baseline data to enable precise evaluation of the role of malaria in eBL in Uganda.

## Methods

### Study setting

**Fig. 1:**
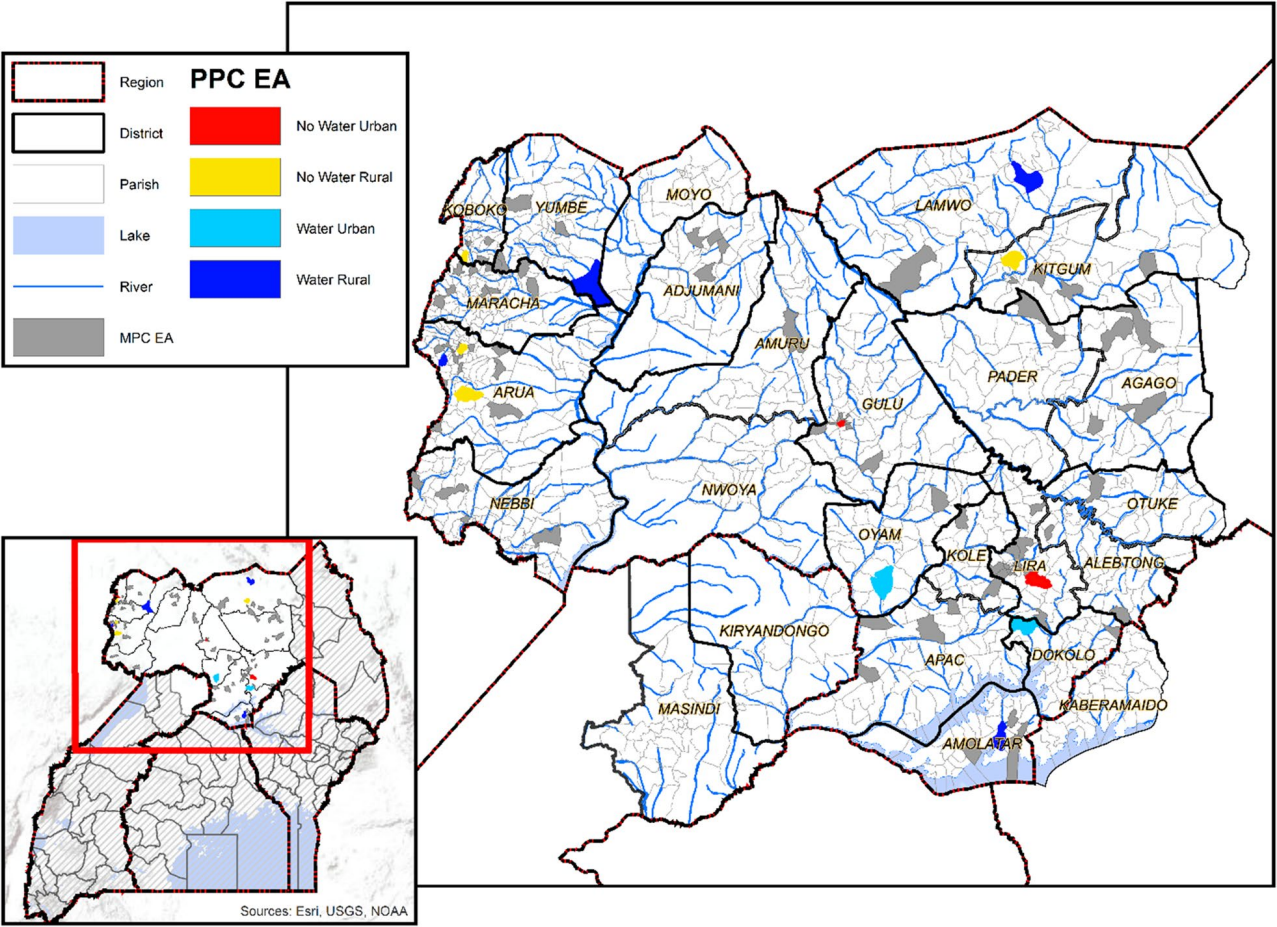
Map of Uganda showing the study area in northern Uganda, including district and census Enumeration area (EA) boundaries. The zoom out shows all-season geographical features, including lakes, rivers or streams. The 12-pilot population control (PPC) EAs are shown according to stratification characteristics (red: near surface water and urban, yellow: far from water and urban, sky blue: near surface water and urban, and azure blue: near water and rural; see “Methods”). The map also shows the 88 EAs that were included in the previous study by Maziarz et al. [13] as gray shaded areas to show their relative geographical distribution in relation to the 12 EAs included in the current study

Between October 2011 and February 2014, a cross-sectional survey of children in 12 random villages in 22 districts Northwest and North-central regions of Uganda (Fig. 1). The children were enrolled as pilot population controls in a case–control study entitled the Epidemiology of Burrkitt Lymphoma in East African children and Minors (EMBLEM) study [13, 18], which is part of a larger study investigating risk factors of eBL in Uganda, Tanzania, and Kenya [19]. The target regions span from 621 to 2000 m above sea level and include areas covered by savannah, slow flowing rivers, swamps, lakes, which are separated into two regions (Northwest and North-central) by a gorge of the East African Rift valley. Both regions experience warm (20–30 °C) and wet climate (1250–1500 mm of rainfall per year [20] in two rainy seasons: April to June and September to December), which is conducive for mosquito breeding and perennial malaria transmission [12]. The malaria prevalence in these regions is high, but geographically heterogenous (prevalence ranging from 3.2 to 75.8% [13]). As noted in the introduction, IRS and LLIN were implemented in 10 districts in the study area between 2008 and 2015 [21], and universal LLIN has been implemented since 2017.

### Sampling design

**Fig. 2:**
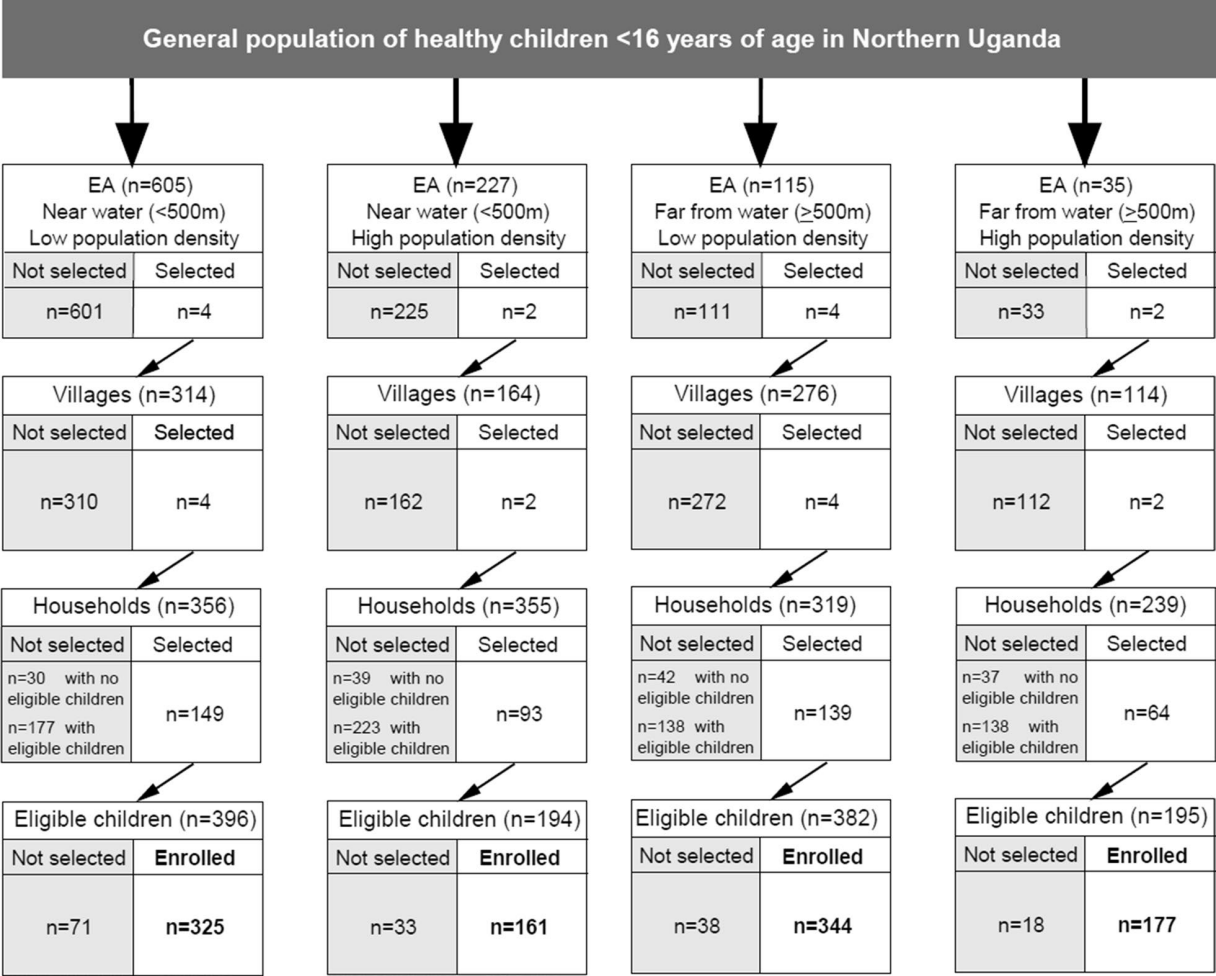
Flow chart showing the stratified, multi-stage cluster sampling design used to sample healthy children aged 0–15 years in 12 randomly selected villages in north-central and northwest regions of Uganda

The target population of children (0–< 16 years old) in northern Uganda was estimated to be 3,031,494 in 2015, based on the national population census data [20]. This population was sampled using a stratified multi-stage cluster sampling design (Fig. 2) [13]. In the first sampling stage, 12 census enumeration areas (EAs) were randomly selected from 4 strata, defined by ‘low population-density’, ‘high population-density’, ‘near water’, and ‘far from water’, from a list of all EAs obtained from the Uganda Bureau of Statistics (UBOS) [20]. These strata are expected to be associated with malaria transmission [22, 23]. Population density of an EA was categorized as low if it was below the median population count (n = 2683), and high if it was equal or higher than the median population count [13]. Proximity of an EA to water was defined as near surface water when the EA boundary was next to or within 500 m of an all-season swamp, river, or lake, based on distances estimated from national maps incorporating geographical information metadata; otherwise, it was defined as being far from water. Four EAs were sampled from the low-density near water stratum and the low density far from water stratum, and 2 EAs from the high density near water stratum and the high density far from water stratum. In the second stage, one village per EA was randomly selected and a household survey conducted, and eligible children in the household invited to participate. Children were eligible if they were aged 0–< 16 years old, were usual residents of the household, and were apparently healthy, i.e., not having symptoms requiring hospital care. Children were not eligible of they had a cancer diagnosis or symptoms requiring hospital treatment.

### Participant enrollment

An experienced field team consisting of laboratory technicians, interviewers, and a local guide, led by a local medical doctor, visited consecutive households in each village to enroll eligible children. For practical reasons, enrollment in each village continued until a sample size of at least 70 children was obtained. The sample size per village was based on a target sample size of 840–1080 children from 12 villages, which was needed to enable precise estimation of population of pfPR in healthy children in the 12 villages as well as in each village. This calculation assumed overall malaria prevalence of 50%, that each village would have at least 30 households with an average household size of 2–3 eligible children, yielding a sample size of 60–90 children per village. Enrollment in each village lasted 2–3 weeks per village, and between October 2011 to February 2014 for the 12 villages. The period of enrollment was prolonged because the study design stipulated that about half of the villages should be enrolled during the dry season and the other half during the wet season. This necessitated phased implementation to enable the single team would be able to mobilize and safely enroll children in all the villages, which were remote, located far away from one another and from the EMBLEM field laboratories (Fig. 1). Written informed permission for the child to take part was obtained from the child’s guardian (usually one or both parents), and assent from children aged 8 years or older. Structured questionnaires were administered to record each child’s age, sex and household characteristics, parental educational level, malaria prevention methods, including ownership and use of LLIN the night before interview and application of IRS in the house, and a history of outpatient or inpatient malaria treatment (≤ 6, 7–12, or ≥ 13 months ago).

### Blood samples and malaria testing

Venous blood for research (10 mL) and clinical (4 mL) tests was drawn in EDTA tubes from each child. The research blood samples were transported in cold boxes to local EMBLEM field laboratories within 2 h from sampling, and centrifuged for 15 min at 1300*g* to separate plasma, buffy coat, and red cell fractions that were stored in barcoded cryovials at − 80 °C. Clinical samples were immediately tested for malaria parasites by experienced local technicians using thick film microscopy (TFM) to visualize asexual malaria parasite forms and commercial rapid diagnostic tests (RDT) (MALARIA DUAL kits, ICT Diagnostics, Muizenberg, Cape Town, South Africa) to detect malaria antigens [13]. TFM was performed on slides stained with 10% Giemsa solution for 10 min, and the visualized parasites were counted against 200 white blood cells (WBCs) and the results expressed as parasites/µL of blood, based on the measured WBC count. The RDT kits used in the current study detect the *P. falciparum*-specific malaria histidine-rich protein 2 (*Pf*-HRP2) and the pan-lactate dehydrogenase (pLDH) antigen and have a reported sensitivity and specificity of 92–100% in Uganda [24]. Because malaria antigens may appear in blood before asexual parasites and remain in blood for several weeks after asexual parasites have been cleared from blood, RDTs were used as the primary outcome measure to capture malaria infection defined by both visualized parasites and circulating antigens [25]. Although RDT tests can yield false-negative results, particularly for parasites that have deleted *pfhrp2* or *pfhrp3* genes that code for the RDT antigens [26], the frequency of such parasites is low (< 1.1) and was considered unlikely to distort the general malaria epidemiological patterns. Thin film smears were examined to identify *Plasmodium* species. Because ~ 98% of the parasites were *P. falciparum*, this species is assumed hereafter.

### Data management

Questionnaires and result forms were reviewed for completeness and accuracy, and computerized by DataFax or computer data entry. DataFax uses intelligent character recognition built-in capabilities to perform data entry and reduce data entry errors. Logical consistency checks were performed on the electronic data, and data queries arising were corrected before creating analysis files.

### Statistical analysis

The response rate was calculated as the proportion of enrolled children out of all eligible children in the selected households that were enrolled. The outcome variable for this analysis was *P. falciparum* prevalence (pfPR), based on RDT positivity, which captures infection defined by visualized asexual parasites and circulating falciparum antigens [25]. Although polymerase chain reaction (PCR) would provide a more accurate result for antigenemia. One child who reported a fever on their questionnaire and had a high parasite count on TFM (> 2500 parasites/uL) was considered to have clinical malaria excluded from analysis. Children who reported a fever on their questionnaire but were malaria negative (n = 6) or had low parasitemia (n = 11, parasite load ranging from scanty to 1200 parasites/uL) were considered to have incidental malaria and not excluded from analysis. The geometric mean parasite density (GMPD) per µL was assessed overall and by age group to confirm low-level parasitemia among children with visualized parasites, as would be expected in high-endemic areas [9].

The results were weighted by the inverse probability of being sampled into the study, and are weighted back to the general population of children aged 0–< 16 years old in Northwest and North-central Uganda in 2015 (projected to be 3,138,360). Weights were trimmed by replacing the value of the weights in the highest 3% of the weight distribution with the value of the weight at the 97th percentile to minimize the impact of outlier weights. Stratification and clustering at the village and household levels were accommodated in the variance computations of estimates. The reported results are weighted estimates, unless stated otherwise.

The analyses were performed using the “survey” package [27, 28] (v. 3.32-1) in R (version 3.4.3, r-project.org) following methods previously described [13]. Descriptive analyses of weighted pfPR are reported overall, by stratum, by village, and by demographical and geographical variables as in [13]. Associations with pfPR were assessed for: (a) household variables reflecting crowding (number of other children in the household), parental characteristics (mother’s income, parents’ occupation), malaria infection in the enrolled household members (any child with infection, infection in a child below 5 years old, infection in a younger sibling); (b) individual characteristics, including history of fever due to malaria or non-malaria conditions (at enrollment, in the past 6 months, in the past 12 months); and (c), use of herbs for treatment of skin or gum disease and lifetime number of admissions. When considering the number of other children in the household or the number of other children in the household below 5 years old, this variable was coded as 0 if there was no other child in the household or no child below 5 years in the household, otherwise it was coded as the number of other children or the number of children below 5 years in the household. Associations between pfPR and keeping different types of animals inside the house or nearby were evaluated because mosquito blood-feeding preferences (anthropophily/zoophily) could influence the risk of malaria positivity [29]. Unadjusted and adjusted odds ratios (ORs), standard errors [30] and Wald-type 95% CIs (95% CIs) [31] of association of pfPR with each variable were calculated. Forward stepwise logistic regression was used to construct adjusted models, using the covariates that in unadjusted models had associations with prevalence with P < 0.05. Covariates with several levels were coded with dummy variables for the categories in the univariate analysis and using trend coding in the adjusted analyses. As the objective of these analyses were descriptive and for hypothesis generation, statistical tests were not adjusted for multiple testing. A two-sided P < 0.05 was considered statistically significant.

## Results

### Demographic characteristics of study population

As shown in Fig. 2, 1007 (86.2%) of 1168 eligible children in 354 (79.5%) of 445 households randomly selected from 1121 eligible households in 12 villages were enrolled. These children represent a trimmed weighted sample of 2,829,988 children aged 0–15 years (Additional file 1: Table S1 and Additional file 2: Fig. S1). The demographic distribution was 45.4% being males and 54.6% females; 42.7% were children aged less than 5 years old, 34.4% aged 5–9 years old, and 23.9% aged 10–15 years old. The size and age distribution of the weighted population were comparable to the corresponding population in the study area in 2015, based on data from UBOS [20].

### Malaria prevalence by rapid diagnostic test and validation by microscopy

Of 1007 enrolled children, 942 (93.5%) were successfully tested for malaria. One subject who reported a fever at enrollment and had parasite load > 2500 parasites/uL was excluded from analysis. In the remaining set, the weighted pfPR was 52.4% (95% CI 19.9–84.8%) by RDT read by experienced local technicians and 32.7% (9.3–56.2%) by TFM performed by experienced local technicians (Table 1). The sensitivity of RDT to detect asexual parasites was 97.5% (95% CI 95.8–99.7%) and the specificity was 69.7% (95% CI 43.4–96.1%), compared to TFM by experienced local technicians.

**Table 1:** Weighted *Plasmodium falciparum* results showing the validation of rapid diagnostic test to detect parasitaemia using thick film microscopy by experienced local technicians, among healthy children in northern Uganda

	Thick film microscopy		Total
	Negative	Positive	
RDT			
Negative	46.9% (n = 391)	0.74% (n = 10)	47.6% (n = 401)
Positive	20.4% (n = 213)	32.0% (n = 327)	52.4% (n = 540)
Total	67.3% (n = 604)	32.7% (n = 337)	100% (N = 941) (N_weighted_ = 2,740,700)

65 subjects who were missing complete malaria data (48 missing both RDT and thick malaria microscopy; 16 missing thick, and 1 missing RDT) were excluded from further analysis. The percentages in each cell are weighted back to the population of size 2,740,700 based on 942 participants with data; the numbers in parentheses are the numbers of individuals with both RDT and thick film microscopy data. Using results from thick malaria microscopy performed by experienced local technicians to validate RDT, the sensitivity was 97.8% (95.8–99.7%) and specificity was 69.7% (43.4–96.1%)

### The overall and age-specific geometric mean parasite density

The overall weighted GMPD was low 733.3 parasites/µL. GMPD was significantly higher in children who were both RDT and TFM positive than those who were TFM-positive but RDT negative (768 versus 107 parasites/µL, *P* = 0.002). GMPD did not vary by sex (P = 0.167), and was not significantly different in children aged below 5 years versus older (955 versus 615 parasites/µL, P_difference_ = 0.201). GMPDs showed modest variation across the villages (189–724 parasites/µL, except for two villages with an average of 1357 and 1807 parasites/µL).

### Malaria prevalence in the 12 villages, by IRS status and season

The weighted pfPR in villages varied from 10.6% in village 19 in the North-central region to 86.2% in village 40 in the northwest region (Table 2). pfPR was lower in IRS villages and higher in non-IRS villages (18.4% versus 75.2%, P < 0.0001), but it varied both within IRS (10.6–34.8%) and non-IRS villages (63.6–86.2%). No overall differences were noted between wet versus dry seasons (66.9% versus 46.2%, P = 0.40). However, within IRS villages, pfPR was lower in villages enrolled in the dry season compared to those enrolled in the wet season (10.6–17.6% versus 20.1–34.8%). No differences in pfPR by season were observed within non-IRS villages (dry: 63.6–75.2% versus wet: 74.8–86.2%; Table 2).

**Table 2:** Weighted prevalence of asymptomatic *Plasmodium falciparum* malaria in 12 random villages in northern Uganda, by season and location in a district where indoor residual insecticide spraying (IRS) was implemented or not

Village	Visit year	Visit months	Season	Region	IRS district	Stratification variables		N enrolled	N with RDT data	Malaria prevalence (%)		Sum of weights for those enrolled (total = 2,829,988)	Sum of weights for those with RDT data (total = 2,763,690)
						Proximity to water	Rural/urban			Unweighted	Weighted		
63	2011	11	Wet	North-central	1	Near	Rural	85	72	16.5	20.1 (10.8, 29.4)	123,080	105,213
75	2014	2	Dry	North-central	1	Near	Urban	88	88	17.0	17.6 (9.6, 25.7)	939,218	939,218
19	2012	2, 3	Dry	North-central	1	Far	Rural	83	80	6.0	10.6 (0.7, 20.4)	30,433	29,382
77	2013	4, 5	Wet	North-central	1	Far	Urban	82	79	28.0	34.2 (20.7, 47.7)	34,195	33,039
29	2012	12	Wet	Northwest	0	Near	Rural	75	75	84.0	79.3 (67.4, 91.2)	355,600	355,600
31	2012	3, 4	Dry, wet	North-central	0	Near	Rural	89	85	61.8	63.6 (48.5, 78.7)	412,313	393,000
45	2013	3	Dry	Northwest	0	Near	Rural	76	71	78.9	85.7 (79.5, 91.9)	203,420	192,167
41	2014	1	Dry	North-central	0	Near	Urban	73	73	75.3	75.2 (63.3, 87.0)	500,210	500,210
100	2012	8, 9	Dry, wet	Northwest	0	Far	Rural	86	82	69.8	74.8 (63.1, 86.5)	76,323	72,991
30	2013	1	Dry	Northwest	0	Far	Rural	84	82	75.0	79.4 (67.8, 90.9)	46,442	44,956
20	2012	6, 7	Wet, dry	Northwest	0	Far	Rural	91	83	71.4	78.5 (68.5, 88.6)	67,092	60,102
40	2011	10, 11	Wet	North-central	0	Far	Urban	94	87	79.8	86.2 (77.6, 94.8)	41,662	37,812

The data in the table are sorted by IRS district, proximity to water (near/far), then by rural/urban. Urban and rural strata defined according to population count in the parish, based on the national census of 2002; proximity of village to water defined as “near” when the parish boundary was < 500 m from an all season surface water body (river, lake, or swamp), otherwise defined as “far”. 1 = January, 2 = February, 3 = March, 4 = April, 5 = May, 6 = June, 7 = July, 8 = August, 9 = September, 10 = October, 11 = November, 12 = December; *NC* North-central, *NW* Northwest, *IRS* indoor residual insecticide spray; dry season months were January to March and July to August; Wet season months were April to June and September to December; The season was deemed to be wet for children enrolled in villages 31 and 100 because most children were enrolled in the wet months, while season was deemed dry for children in village 20 because most were enrolled in the dry season month; Weights were trimmed to the 97th percentile, affecting 30 children whose weight was set to 14,409.34

### pfPR and geometric mean parasite density, by population density strata, season, age

**Fig. 3:**
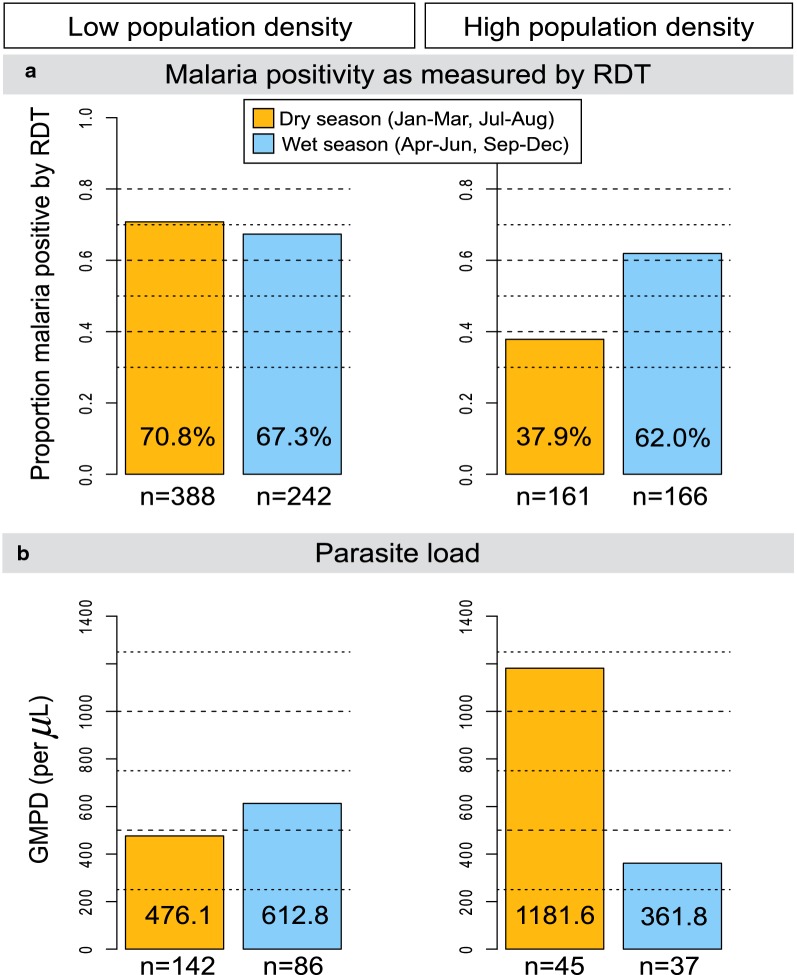
Bar graphs showing weighted malaria weighted per cent *Plasmodium falciparum* parasite prevalence, based on the RDT, by season (wet or dry) in low- and high-population density villages (**a**) and the GMPD/µL among microscopy-positive children by visit season in low- and high-population density villages (**b**) among apparently healthy children enrolled in 12 random between November 2011 and February 2014 in north-central and northwest Uganda. Note: Orange shading is used for dry season months, while blue shading is used for wet season months. Wet and dry seasons are based on categorization by the Uganda Bureau of Statistics and generally correspond to ≥ 10 days/month for wet months and < 10 days/month for dry months. The unweighted number of participants in each group is shown

The stratum-specific weighted pfPR by season are shown in Fig. 3. Within the low-population density stratum, pfPR in the wet and dry season was similar (67.3% versus 70.8%, P = 0.802). Conversely, within the high-population density stratum, pfPR was higher in the wet season than in the dry season, but not statistically significant (62.1% versus 37.9%; P_heterogeneity_ = 0.589; Fig. 3a). GMPD did not vary by season in the low- or high-population density strata (Fig. 3b), but the GMPD tended to be higher in the dry season than the wet season in the high-density stratum.

**Fig. 4:**
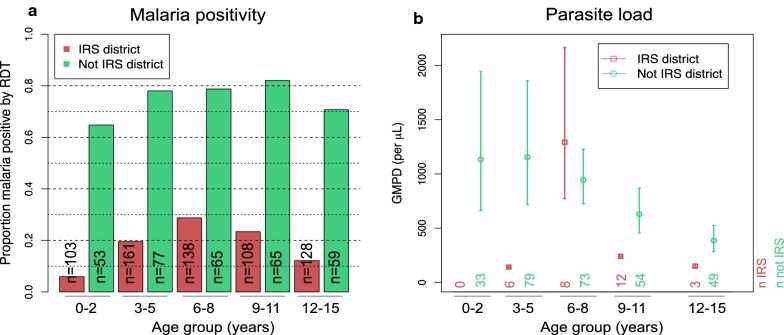
Bar graphs showing age-group patterns of weighted *Plasmodium falciparum* parasite prevalence, based on RDT, stratified by whether the village is in a district where indoor residual insecticide spraying (IRS) against mosquitoes was implemented (IRS district: red color) or not (non-IRS district: green color) (**a**) and the GMPD parasites/µL (**b**). Results are from apparently healthy, microscopy-positive children enrolled in 12 randomly selected villages between October 2011 and February 2014 in northern Uganda. The unweighted number of participants in each age-group is shown. In **b**, the open circle, or rectangles shows the GMPD parasites/µL, the lines show the 95% CIs of the GMPD. GMPD results were available on 328 with positive results of 339 (includes 1 subject who was RDT negative) tested by thick film microscopy; thick film negative subjects were not included (see Table 1)

The age-specific pfPR was higher in non-IRS versus IRS villages, but slight differences in the actual peaks. pfPR peaked between 6 and 8 years old in IRS villages and slightly later between 9 and 11 years old in non-IRS villages (Fig. 4a). Among children with visualized parasites, the age-specific-GMPD was higher in non-IRS villages (Fig. 4b), in children up to 6–8 years of age and then decreased rapidly in older children. The GMPD in children in IRS villages was very low for all ages, although a spike was observed in children aged 6–8 years old (Fig. 4b), probably due to random fluctuation.

### Household- and individual-characteristics associations with weighted pfPR in children

The associations between pfPR and children’s demographical, malaria prevention, illness history, lifetime malaria treatment, environmental, and household characteristics are shown in Table 3. No differences in pfPR were noted between females and males (53.3% versus 51.4%; P = 0.768), by distance of the home from the source of drinking water being ≥ 1 or < 1 km (55.7% versus 49.4%; P = 0.723). pfPRwas associated with keeping goats inside or near the house (49.8% versus 60.5%, OR 0.65, 95% CI 0.48–0.87; *P *= 0.021).

**Table 3:** *Plasmodium falciparum* parasite prevalence (pfPR) among children 0–15 years old enrolled between October 2011 and Feb 2014 in 12 villages in north-central and northwest regions of Uganda and associations with sex and those characteristics that resulted in P < 0.05 in univariate logistic models

Characteristics	n positive	Unadjusted		P*	Adjusted	P*
		Weighted pfPR %	Odds ratio (95% CI)		pfPR odds ratio (95% CI)^¶^	
All subjects	553	52.5	–	–	–	–
Sex						
Female	287	53.3	Ref.			
Male	266	51.4	0.93 (0.56–1.52)	0.767	–	–
Mother’s income (Ugandan shillings)						
< 30,000 USHS	242	47.9	Ref.			
≥ 30,000 USHS	309	57.7	1.48 (1.09–2.01)	*0.036*	–	–
Distance of home to water source						
≥ 1 km	338	55.7	Ref.			
< 1 km	215	49.4	0.78 (0.20–3.00)	0.723	–	–
Malaria prevention						
Indoor residual spraying (IRS) sub- region						
Not an IRS district	496	75.2	Ref.		Ref.	
IRS district	57	18.4	0.07 (0.05–0.11)	*< 0.0001*	0.06 (0.04–0.07)	*< 0.0001*
Indoor residual spraying (IRS) in house						
Never	506	59.0	Ref.			
In the past year	47	23.7	0.22 (0.05–0.86)	0.061	–	*–*
Mosquito net used last night						
No	391	48.8	Ref.			–
Yes	162	64.6	1.91 (0.46–7.89)	0.395	–	
Number of other children in household						
1	58	67.9	Ref.		Ref.	
2	81	43.6	0.37 (0.22–0.60)		0.70 (0.69–0.73)	
3	98	50.9	0.49 (0.35–0.68)		0.49 (0.45–0.52)	
4	110	40.6	0.32 (0.21–0.49)		0.34 (0.31–0.37)	
5	88	61.6	0.76 (0.57–1.00)		0.24 (0.21–0.26)	
6+	118	54.4	0.56 (0.24–1.31)	*< 0.0001*	0.17 (0.15–0.19)	*< 0.0001*
Number of children below 5 years in household						
0	130	55.5	Ref.		Ref.	
1	268	58.6	1.14 (0.75–1.72)		0.48 (0.30–0.76)	
2–4	155	36.5	0.46 (0.32–0.66)	*0.0002*	0.23 (0.12–0.44)	*0.014*
Kept goat near or inside house						
No	160	60.5	Ref.		Ref.	
Yes	393	49.8	0.65 (0.48–0.87)	*0.021*	0.42 (0.29–0.62)	*0.0002*
Non-malaria fevers						
In past 6 months before enrollment						
No	461	51.4	Ref.			
Yes	92	60.6	1.45 (0.66–3.21)	0.381		
Household members with malaria						
Any child positive with malaria						
None	58	29.6	Ref.			
≥ 1 positive	495	64.1	4.24 (1.61–11.2)	*0.019*		
Younger sibling positive with malaria						
No	266	46.0	Ref.		Ref.	
Yes	287	65.1	2.20 (1.40–3.45)	*0.009*	5.39 (2.94–9.90)	*0.0006*
Malaria fever						
Reports a fever at enrollment						
No	542	52.2	Ref.			
Yes	12	85.5	5.38 (2.01–14.4)	*0.010*	4.80 (1.94–11.9)	*0.0094*
Fever count in past 6 months before enrollment						
0	132	44.7	Ref.			
1	113	42.1	0.90 (0.59–1.38)			
2	101	54.7	1.50 (0.83–2.71)			
3	102	66.0	2.40 (0.82–7.05)			
4+	105	69.4	2.80 (1.11–7.05)	*0.0003*		
In past 12 months before enrollment						
No	80	38.3	Ref.			
Yes	462	55.7	2.02 (1.25–3.27)	*0.021*	–	
Lifetime malaria treatment						
Inpatient						
Past 12 months	135	65.1	Ref.			
More than 12 months	114	57.4	0.72 (0.25, 2.09)			
Never	304	46.6	0.47 (0.13, 1.73)	*0.027*		
Outpatient						
Past 12 months	395	53.3	Ref.			
More than 12 months	76	67.6	1.83 (1.15, 2.92)			
Never	82	39.6	0.57 (0.41, 0.80)	*0.0057*		

* Covariates with several levels were coded with dummy variables for the categories and P is for heterogeneity) in the univariate analysis and using trend coding in the adjusted analyses (P is for trend). ^¶^ Final adjusted models used forward stepwise regression starting with 14 variables with P < 0.05 in univariate models (IRS district, mother’s income, number of other children in the household, number of malaria fevers in the past 6 months, having a younger sibling with malaria, keeping a goat in the house, inpatient and outpatient treatment for malaria). Mother’s income was estimated in Ugandan shillings (30,000 Ugandan shillings are approximately equal to 10 US dollars). The survey estimates are weighted estimates that account for the differential probabilities in selecting the sample of children. Variance estimation takes the weights into account and accounts for the clustering of the sample of children at the village and household levels. The coefficient of variation of the final weights was 1.25 (defined as standard deviation/mean of the final weights)

Compared to living in a non-IRS village, living in an IRS village was associated with decreased pfPR (18.5% versus 75.2%, OR 0.07, 95% CI 0.05–0.11). Consistent with this result, pfPR was decreased in children for whom their questionnaire reported that IRS was applied to their house, as compared to no application (23.7% versus 59.0%, OR 0.22, 95% CI 0.05–0.86). Consistent with previous results [13], sleeping under an LLIN on the night before enrollment, versus not, was not associated with decreased pfPR (64.6% versus 48.8%, *P *= 0.395). Compared to having no other child in the household, having one or more other children in the household, as was associated with decreased pfPR (P_heterogeneity_ < 0.0001; Table 3). The results were similar when the number of children below 5 years old was considered (P_heterogeneity_ = 0.0002).

Reporting a history of non-malaria related fever in the past 6 months, versus not, was unrelated to pfPR (60.6% versus 51.4%, *P *= 0.384). However, reporting four or more malaria-related fever episodes in the past 6 months, versus none, was significantly associated with elevated pfPR (OR 2.80, 95% CI 1.11–7.05, P_heterogeneity_ = 0.0003). Compared to children reporting a history of inpatient malaria treatment in the past 12 months, pfPR was lower in those reporting a history of inpatient malaria more than 12 months ago (OR 0.72) and those reporting no such a history ever (OR = 0.47, P_trend_ = 0.026; Table 3). In contrast, compared to children reporting a history of outpatient malaria treatment in the past 12 months, the pfPR was elevated in those reporting a history of outpatient treatment of malaria more than 12 months ago (OR 1.83, 95% CI 1.15–2.91), but decreased in those reporting no such history ever (OR 0.57, 95% CI 0.41–0.80, Table 3).

A few children (n = 19) who reported a fever on their questionnaire were deemed to have incidental malaria at the time of enrollment. Compared to those who did not report a fever, reporting a fever was associated with pfPR (OR 5.38, 95% CI 2.01–14.39). pfPR was associated with having at least one sibling who was RDT positive (versus none: OR 4.24 95% CI 1.61–11.15) and with having a younger sibling who was RDT positive (versus none: OR 2.20 95% CI 1.40–3.46). Season, parental education and occupation, herbal treatment, and keeping other animals (other than goat) inside or near the house was not associated with pfPR (Table 4).

**Table 4:** Patterns of *P. falciparum* parasite prevalence according to environmental, parental, and household characteristics in children aged 0–15 years old enrolled in 12 villages in north-central and northwest regions of Uganda between October 2011 and Feb 2014

	Number positive	Weighted pfPR %	OR 95% CI			*P*
Environment/home characteristics						
Season						
Dry season	304	46.2	Ref.			
Wet season	249	66.9	2.35	0.35–15.7		0.404
Home location						
Urban	340	42.8	Ref.			
Rural	211	67.5	2.77	0.37–20.9		0.350
Parents education/occupation						
Mother’s education level						
Up to primary 4	315	53.5	Ref.			
Primary 5 or higher	236	51.5	0.92	0.64–1.34		0.685
Mother’s occupation						
Other	40	57.2	Ref.			
Subsistence farmer	511	52.2	0.81	0.36–1.85		0.637
Father’s education level						
Up to primary 4	136	61.9	Ref.			
Primary 5 or higher	415	50.6	0.63	0.36–1.11		0.147
Father’s occupation						
Other	103	47.6	Ref.			
Subsistence farmer	448	53.6	1.27	1.04–1.56		0.050
Keeping animals near or inside the house						
Chicken						
No	106	45.2	Ref.			
Yes	447	54.2	1.43	0.52–3.92		0.505
Pig						
No	473	51.8	Ref.			
Yes	80	56.3	1.20	0.29	4.94	0.809
Sheep						
No	393	48.3	Ref.			
Yes	160	61	1.68	1.05	2.69	0.064
Cow						
No	304	47.4	Ref.			
Yes	248	56.5	1.44	0.95	2.18	0.121
Birds						
No	462	52.5	Ref.			
Yes	91	52.7	1.01	0.71	1.44	0.958
Dog						
No	349	49.4	Ref.			
Yes	204	57.4	1.38	0.65	2.93	0.430
Use of herbs for treatment						
Herb applied to skin						
No	427	51.5	Ref.			
Yes	126	61.5	1.50	0.67	3.38	0.352
Herb applied to gums						
No	292	49.8	Ref.			
Yes	261	59.4	1.47	0.51	4.21	0.492
Number of hospital admissions since birth						
0	267	47.3	Ref.			
1	131	54.1	1.31	0.92	1.86	
2	58	62.9	1.89	0.40	9.01	
3+	97	61.2	1.75	0.61	5.03	0.402

OR, odds ratio; 95% CI, 95% confidence intervals

Only 6 variables of 14 evaluated using stepwise logistic regression remained significantly associated with pfPR in the multivariable model. Specifically, pfPR was inversely associated with living in an IRS village (adjusted OR 0.06, 95% CI 0.04–0.07), with having two or more other children in the household (versus none, P_trend_ < 0.0001), or 2–4 children in the household aged below 5 years of age (versus none, P_trend_ = 0.014), and keeping a goat inside or near the house (aOR 0.42, 95% CI 0.29–0.62). Conversely, pfPR was positively associated with reporting a fever at enrollment (aOR 5.02, 95% CI 1.95–12.92) and having at least one sibling who was RDT positive (aOR 5.39, 95% CI 2.94–9.90).

## Discussion

The current analysis, conducted to generate recent village, household, and individual-level data about incidental malaria infections in children living in 12 villages in northern Uganda, confirms the high overall pfPR in the villages studied. Similar to findings based on a study of children in 100 villages [13], the study shows substantial heterogeneity on pfPR in different villages. The study also confirmed that IRS was major factor driving pfPR patterns, but noted substantial heterogeneity both within IRS and non-IRS villages. Further research is needed to elucidate factors influencing malaria heterogeneity in villages in Uganda, regardless of their IRS status. The pfPR patterns in non-IRS villages did not vary by season suggesting that the prevalence of incidental malaria is saturated in the dry and wet season due to perennial transmission and a high level of herd immunity [32]. Conversely, although not statistically significant, the pfPR in IRS villages increased two-fold from dry to wet season. This pattern suggests that the vector-host ratio in the IRS district villages increases during the wet season [33]. This pattern suggests that malaria prevalence increases in the wet season, presumably due to a corresponding increase in the vector-host ratio, and predicts that malaria prevalence will increase sharply during the wet season when IRS is withdrawn. Consistent with this, malaria epidemics were reported in northern Uganda following the withdrawal of IRS in 2014 [15], and the sharpest increases were noted during the wet seasons. The results presented here suggest that preemptive IRS before onset of the wet season or in areas where IRS has been withdrawn could offer some benefits to the population.

The use of insecticide-treated bed nets the night before interview was not associated with pfPR, confirming the results reported by other studies conducted in northern Uganda [13, 34] and elsewhere [35]. This finding may be pointing to the emergence of mosquito vector metabolic resistance to pyrethroids impregnated into bed nets [36] and a threat against the continued effectiveness of insecticide-treated bed nets in the fight against malaria [37, 38]. These results call for careful monitoring of malaria case activity at a district level and proactive correlation of uptake of universal distribution of insecticide-treated bed nets with clinical case activity, such as using electronic health management information systems (eHMIS) [39]. The results also point to a need for more research to investigate pyrethroid resistance and alternative chemicals if insecticide-treated bed nets are to be maintained as a key malaria control tool in malaria endemic countries [5, 6].

The interest in malaria patterns in northern Uganda had an addition motivation. Malaria is thought to be important in eBL [18], and a better understanding of its patterns in needed to improve the design of the EMBLEM study [40], and its goal to understand and control for geographical patterns of eBL [41]. The finding of mostly low-grade malaria parasitaemia in the BL-age children in this study is consistent with the hypothesis that children at risk of eBL generally have a high level of anti-parasite immunity [42]. If so, the baseline risk factors for pfPR (low grade malaria parasitaemia) in children with established immunity may be relevant for investigating and interpreting malaria-related risk factors for eBL, particularly in northern Uganda [43, 44]. For example, the total number of other children (or those below 5 years) living in a household was inversely associated with prevalence of low-grade malaria infection would be hypothesized to have an inverse relationship with eBL. Conversely, having a child with confirmed malaria infection in the household was associated with low-grade malaria infection, may confound associations with eBL. The finding that keeping goats inside or near the house was inversely associated with pfPR was unexpected, but interesting. This finding may be a clue about the role of zooprophylaxis or zoopotentiation [29], due to feeding preference of mosquito vectors for humans of animals [29], influencing malaria risk. Donnelly et al. [45] reported that keeping goats or chicken inside the house was significantly associated decreased risk of malaria in Kanungu District in Western Uganda. Bulterys et al. [46] reported that a goat: human ratio ≥ 1 was associated with reduced the risk of malaria infection in a study conducted in a village in Zambia. These findings suggest that keeping of certain animals inside or near the house may influence malaria risk, perhaps, by drawing vectors away from humans. Taken together, these results will enable hypothesis-driven research about factors associated with eBL risk in northern Uganda, and elsewhere.

The limitations of the study include reliance on cross-sectional data and lack of geospatial data to complement questionnaire data. The lack of recent entomological data limits the discussion about abundant vectors in northern Uganda, and specifically about feeding preference of the vectors prevalent in northern Uganda and whether their relative distribution has changed from before to after the implementation of malaria suppression [21]. Surveys conducted in the 1960s and 1970s suggested the abundant species as *Anopheles gambiae* and *Anopheles funestus*, which are antropophilic [47], while *Anopheles arabiensis*, which is feeds both on humans and animals was less abundant [48], but the current patterns are unknown.

## Conclusions

The study confirmed high, but heterogenous asymptomatic pfPR in children in 12 villages in northern Uganda. The pfPR was strongly influenced by IRS, but showed substantial variation both within IRS and non-IRS villages. pfPR was inversely associated with living in an IRS village, having a greater number of other children, or children aged below 5 years of age in the household, and keeping a goat inside or near the house. Conversely, pfPR was positively associated with reporting a fever at enrollment and having at least one sibling who was RDT positive. Further research is needed to elucidate factors influencing malaria heterogeneity in villages in Uganda, and to determine the value of developing public health messages that stress household characteristics associated with pfPR.

## Additional files

**Additional file 1: Table S1.** Weighted distribution of characteristics of apparently healthy children aged 0–15 years enrolled between October 2011 and February 2014 in 12 villages in northern Uganda.

**Additional file 2: Fig. S1.** Age distribution in single year age groups of the unweighted and weighted population of the children enrolled in 12 randomly selected villages in northern Uganda.

## Abbreviations

pfPR: *Plasmodium falciparum* prevalence
*pfhrp2*: *Plasmodium falciparum* histidine-rich protein 2
*pfhrp3*: *Plasmodium falciparum* histidine-rich protein 3
GMPD: geometric mean parasite density
OR: odds ratio
CI: confidence interval
AEIR: annual entomological inoculation rate
BL: Burkitt lymphoma
IRS: indoor residual spraying
EA: enumeration area
UBOS: Uganda Bureau of Statistics
TFM: thick film microscopy
WBC: while blood cell
RDT: rapid diagnostic test
pLDH: pan lactate dehydrogenase
USHS: Ugandan shillings
US$: United States dollar
