# Supplementary material for: A cross-sectional study of asymptomatic Plasmodium falciparum infection burden and risk factors in general population children in 12 villages in northern Uganda
Source: Malar J. 2018 Jun 20;17:240. doi: 10.1186/s12936-018-2379-1 (PMC6011516; doi:10.1186/s12936-018-2379-1)
Supplement: Supplementary file 1 — Additional file 1: Table S1. Weighted distribution of characteristics of apparently healthy children aged 0–15 years enrolled between October 2011 and February 2014 in 12 villages in northern Uganda. [file 12936_2018_2379_MOESM1_ESM.docx]

Additional Table S1: **Weighted distribution of characteristics of apparently healthy children aged 0-15 years enrolled between October 2011 and February 2014 in 12 villages in northern Uganda**

|  |  | Weighted | |  |
| --- | --- | --- | --- | --- |
|  | **N=1006** | **%** | **SE*** |  |
| **Age group, years** |  |  |  |  |
| 0-5 | 436 | 42.7 | 2.1 |  |
| 6-10 | 326 | 33.4 | 1.6 |  |
| 10-15] | 244 | 23.9 | 1.5 |  |
| **Sex** |  |  |  |  |
| Female | 519 | 54.6 | 1.6 |  |
| Male | 487 | 45.4 | 1.6 |  |
| **Proximity to water** |  |  |  |  |
| Far (>500m) | 520 | 10.4 | 2.3 |  |
| Near (< 500m) | 486 | 89.6 | 2.3 |  |
| **Population density** |  |  |  |  |
| Low (<2,683 children) | 669 | 46.4 | 8.9 |  |
| High (>2,683 children) | 337 | 53.6 | 8.9 |  |
| **Season** |  |  |  |  |
| Dry season | 567 | 69 | 10.4 |  |
| Wet season | 439 | 31 | 10.4 |  |
| **Region** |  |  |  |  |
| North-central | 594 | 73.5 | 12.8 |  |
| Northwest | 412 | 26.5 | 12.8 |  |
| **IRS district** |  |  |  |  |
| ~~~ Not an IRS district | 668 | 60.2 | 28.1 |  |
| ~~~ IRS district | 338 | 39.8 | 28.1 |  |
| **Indoor residual spraying (IRS) in house** |  |  |  |  |
| Never | 733 | 81.3 | 11.1 |  |
| In the past year | 272 | 18.7 | 11.1 |  |
| **Sub regions** |  |  |  |  |
| 1 | 159 | 14.2 | 12.4 |  |
| 2 | 253 | 12.3 | 8.4 |  |
| 4 | 85 | 4.3 | 4.7 |  |
| 5 | 333 | 66.6 | 12.5 |  |
| 6 | 82 | 1.2 | 1.2 |  |
| 7 | 94 | 1.4 | 1.5 |  |
| **Mother's education** |  |  |  |  |
| Up to primary 4 | 537 | 49.3 | 3.7 |  |
| Primary 5 or higher | 466 | 50.7 | 3.7 |  |
| **Mother's income (Ugandan shillings)** |  |  |  |  |
| <30,000 USHS | 481 | 53.4 | 4.6 |  |
| ≥30,000 USHS | 522 | 46.6 | 4.6 |  |
| **Mosquito net used last night** |  |  |  |  |
| No | 744 | 76.1 | 8.2 |  |
| Yes | 261 | 23.9 | 8.2 |  |
| **Inpatient for malaria** |  |  |  |  |
| In past 12 mths | 254 | 20.9 | 6.7 |  |
| More than 12 months ago | 207 | 19.5 | 3.4 |  |
| No | 544 | 59.6 | 8.6 |  |
| **Outpatient for malaria** |  |  |  |  |
| In past 12 mths | 733 | 69.9 | 6.1 |  |
| More than 12 months ago | 119 | 11.5 | 2.9 |  |
| No | 153 | 18.6 | 8 |  |
|  |  |  |  |  |
|  |  |  |  |  |
| **Note**: N shows unweighted numbers; Dry season months were January to March and July to August; Wet season months were April to June and September to December. Mother’s income was estimated in Ugandan shillings (30,000 Ugandan shillings are approximately equal to 10 US dollars). The survey estimates are weighted estimates that account for the differential probabilities in selecting the sample of children (see Methods). Variance estimation takes the weights into account and also accounts for the clustering of the sample of children at the village level. * SE are large due to high intra-class correlation within village. | | | | |
|  |  |  |  |  |
