# Supplementary material for: A cross-sectional study of asymptomatic Plasmodium falciparum infection burden and risk factors in general population children in 12 villages in northern Uganda
Source: Malar J. 2018 Jun 20;17:240. doi: 10.1186/s12936-018-2379-1 (PMC6011516; doi:10.1186/s12936-018-2379-1)
Supplement: Supplementary file 2 — Additional file 2: Fig. S1. Age distribution in single year age groups of the unweighted and weighted population of the children enrolled in 12 randomly selected villages in northern Uganda. [file 12936_2018_2379_MOESM2_ESM.docx]

**Additional file 2: Fig. S1 Age distribution in single year age groups of the unweighted and weighted population of the children enrolled in 12 randomly selected villages in northern Uganda.**
